# Gelatinous drop-like corneal dystrophy with a novel mutation of *TACSTD2* manifested in combination with spheroidal degeneration in a Chinese patient

**Published:** 2010-08-11

**Authors:** Bei Zhang, Yu-Feng Yao

**Affiliations:** Department of Ophthalmology and Sir Run Run Shaw Institute of Clinical Medicine, Sir Run Run Shaw Hospital, Zhejiang University School of Medicine, Zhejiang, P.R. China

## Abstract

**Purpose:**

To report the clinicopathological findings of a Chinese patient with an unusual phenotype of gelatinous drop-like corneal dystrophy (GDLD) combined with spheroidal degeneration and to detect molecular defect in the tumor-associated calcium signal transducer 2 (*TACSTD2*) gene.

**Methods:**

Extensive physical and ophthalmologic examination of the patient was performed. Initially superficial keratectomy was performed for both eyes. Due to recurrence of the corneal opacity, penetrating keratoplasty for the right eye and deep lamellar keratoplasty for the left eye were performed. The obtained corneal tissues were examined by light microscopy. Molecular genetic analysis consisted of PCR amplification and direct automated sequencing of the complete coding region of *TACSTD2*.

**Results:**

Slit-lamp biomicroscopy of the patient revealed bilateral band-like corneal opacities composed of brown-yellow, oily appearing droplets at the first visit. Two years after superficial keratectomy, elevated mulberry-like gelatinous lesions companied with brown-yellow droplets in the superficial cornea in both eyes were found. Histological analysis of corneal tissue revealed subepithelial amorphous deposits stained positively with Congo red, typical of GDLD. Meanwhile, eosinophilic globular deposits with irregular peripheral margins and various sizes, which were characteristics of spheroidal degeneration, were found. Sequencing of *TACSTD2* from the patient revealed a novel homozygous missense mutation c.354G>C, leading to amino acid substitution Q118H in the patient.

**Conclusions:**

This is the first report indicating a new type of gelatinous drop-like corneal dystrophy (GDLD) combined with spheroidal degeneration. Molecular analysis demonstrated a novel mutation in *TACSTD2*, which may expand the spectrum of mutations in *TACSTD2*.

## Introduction

Gelatinous drop-like corneal dystrophy (GDLD, OMIM 204870) is a rare autosomal recessive corneal dystrophy, most often found in Japan [1]. Clinical manifestations usually appear in the first decade of life with bilateral, axial, elevated, mulberry-like gelatinous lesions, due to primary amyloid deposition in the subepithelium and anterior stroma. The deposits spread laterally and deeply within the stroma with time, leading to a progressive loss in vision, photophobia, and foreign-body sensation [2]. The gene responsible for GDLD was tumor-associated calcium signal transducer 2 (*TACSTD2*) located at chromosome 1p32 [3].

Spheroidal degeneration is also a rare corneal disease characterized by a band-shaped pattern of brown-yellow, globular, oily appearing deposits measuring 0.1–0.6 mm in diameter in the subepithelium, and superficial corneal stroma. The lesions are first seen at the limbus in the 3 and 9 o’clock positions and are restricted to the interpalpebral zone, which may enlarge and spread toward the central cornea progressively [4]. There are various alterative names for spheroidal degeneration in the literature, including Bietti’s nodular corneal degeneration, Labrador keratopathy, climatic droplet keratopathy, fisherman’s keratopathy, and Eskimo corneal degeneration. Spheroidal degeneration is especially common in parts of the world with high levels of exposure to ultraviolet radiation. The exact etiologic and pathologic nature remains controversial [5]. Light microscopy reveals extracellular eosinophilic globular deposits of variable size in the subepithelium and anterior stroma. The epithelium varied in thickness. The spheroidal deposits stained green to black with Verhoeff-van Gieson, brown with Masson’s trichrome, and negatively for Congo red and periodic acid–Schiff (PAS). Special stains did not reveal any iron or calcium in the spheroids [6].

In the present study we report an unusual case of gelatinous drop-like corneal dystrophy (GDLD) combined with spheroidal degeneration. In addition, we identified a novel mutation, using molecular genetic analysis, in *TACSTD2*.

## Methods

### Subjects

A 22-year-old ethnic Chinese man was first referred to our hospital in 2000. He experienced a slowly progressive loss of vision accompanied with photophobia and lacrimation of both of his eyes beginning at the age of 17. There was no history of excessive exposure to adverse environmental conditions and ocular trauma. He was born of a normal pregnancy to a non-consanguineous family in Zhejiang province in the central regions of China. The patient was in good health and physical examination showed no other abnormality. He was single and had one brother. Ophthalmic examination of his parents and brother did not reveal any ocular abnormalities, and there were no known similar ocular conditions in the family.

The study was approved by the institutional ethics committee of Sir Run Run Shaw Hospital. Informed consent was obtained from the patient and their family members who participated in this study.

### Histological examination

Corneal tissues were fixed immediately in 3% formalin for light microscopy, using a standard protocol, following surgery. Specimens were stained with hematoxylin and eosin, periodic acid Schiff, Masson trichrome, and Congo red for light microscopic evaluation.

### Mutation analysis

Molecular genetic analyses were performed in the patient and his parents. Fifty normal Chinese subjects were enrolled as control. Genomic DNA was extracted from peripheral blood by standard procedures. The exon of *TACSTD2* was amplified using 3 sets of primers (Table 1) to generate overlapping products, which were screened for mutations by direct sequencing [7].

**Table 1:** *TACSTD2* primer sequences.

**Primer**	**Sequence (5’-3’)**	**Product size (bp)**	**Annealing temperature (°C)**
F1	ACGTGTCCCACCAACAAGAT	681	60
R1	CAGGTAATAGATGAGCGTGCG		64
F2	GGATGTGTCACCCAAATACCA	423	62
R2	CTTGAGCAGCAGACACTTGGA		64
F3	CCTACTACTTCGAGAGGGACA	382	64
R3	CAGGAAGCGTGACTCACTT		58

Polymerase chain reaction (PCR) was performed in a volume of 50 µl mixture containing 1 µM of each primer, 0.5 U of Hotstar Taq polymerase (Qiagen, Hilden, Germany), 250 µM dNTP mixture, 5 µl of 10× PCR Buffer with MgCl_2_, 10 µl of 5× Q-solution (Qiagen), and approximately 100 ng of human genomic DNA. Thermal cycling was performed using a GeneAmp PCR System 9700 (Applied Biosystems, Foster City, CA) with the following program: 15 min at 95 °C, followed by 32 cycles of 94 °C for 1 min, 60 °C for 1 min and 72 °C for 1 min, with a final extension step at 72 °C for 7 min.

Amplified DNA was purified using the QIAquick PCR purification kit (Qiagen) and sequenced according to the protocols accompanying the BigDye Terminator cycle sequencing kit (Applied Biosystems). An ABI Prism 377 Genetic Analyzer (Applied Biosystems) was used to collect and analyze the sequence data. DNA was sequenced in both the forward and reverse directions.

## Results

### Clinical manifestations and therapeutic course

**Figure 1:**
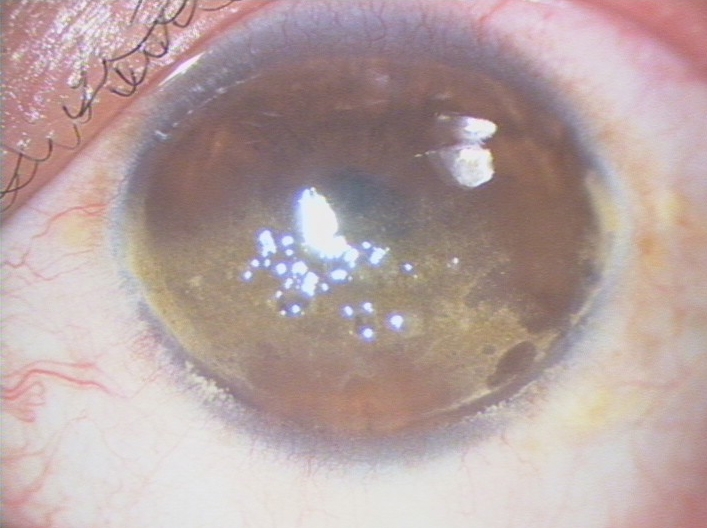
Slitlamp photograph of the right cornea of the proband at the first visit. There are band-like intrapalpebral corneal opacities in the superficial layers of the cornea. The opacities are composed of brown-yellow, globular, oily appearing droplets that tend to be confluent and elevate the epithelium.

On his first visit in January 2000, the best-corrected visual acuity of the proband was 6/15 in the right and 6/30 in the left eye. Slit-lamp examination revealed bilateral, symmetric band-like corneal opacities occupying the region of the palpebral fissure of both eyes (Figure 1). The opacities were composed of brown-yellow, globular, oily appearing droplets, inclining to be confluent and elevated the epithelium. All these deposits were limited to superficial layers of the corneal stroma. The deeper layers of the stroma, Descemet’s membrane and the endothelium were normal. There was mild injection of bulbar conjunctivae. A clinical diagnosis of corneal spheroidal degeneration was made.

**Figure 2:**
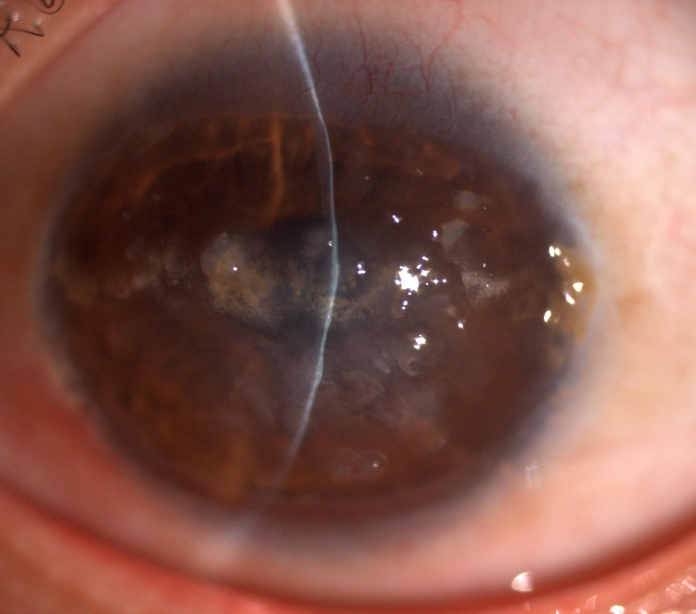
Slitlamp photographs of the right cornea of the proband two years after the superficial keratectomy. There is diffuse corneal opacity with multiple grayish-white nodular elevations companied with yellowish globular droplets in the anterior stroma.

**Figure 3:**
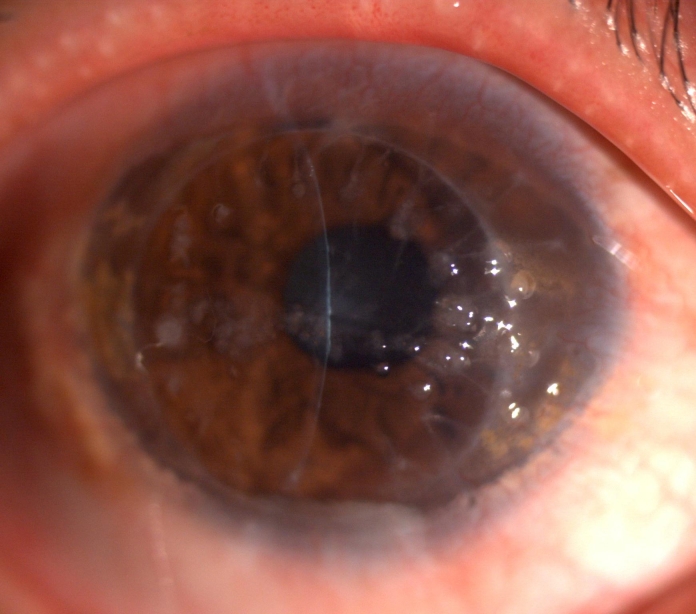
Slitlamp photographs of the right cornea of the proband four year after penetrating keratoplasty. Elevated, mulberry-like gelatinous lesions companied with yellowish globular droplets are showed.

The proband was treated by superficial keratectomy in both eyes in January 2000. The vision was improved to 6/12 for one year and then decreased. Slit-lamp examination showed grayish-white nodular elevations companied with yellowish globular droplets in the anterior stroma in both eyes two years after the surgery (Figure 2). A clinical diagnosis of gelatinous drop-like corneal dystrophy (GDLD) combined with spheroidal degeneration was made. In March 2004, the best-corrected visual acuity of the proband was 6/24 in both eyes and a penetrating keratoplasty was performed in the right eye. In July 2008, a deep lamellar keratoplasty was performed in the left eye. Both eyes restored corneal clarity and achieved a best corrected visual acuity as 6/12 at 1 year postoperatively. There was obvious recurrence of corneal opacity in the right eye four years later and the vision acuity decreased to 6/30 in 2009. Slit-lamp examination showed elevated, mulberry-like gelatinous lesions companied with yellowish globular droplets (Figure 3). There was no obvious recurrence of corneal opacity in the left eye till now.

### Histological examination

**Figure 4:**
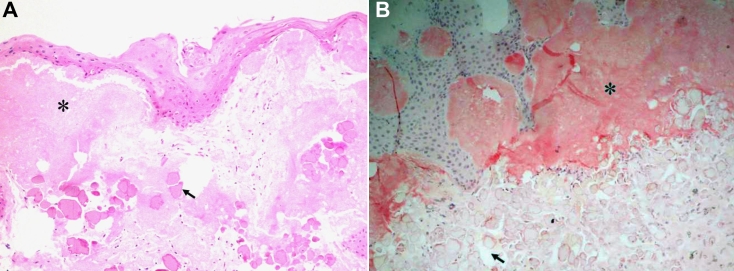
Histopathological findings of the corneal button taken from the right eye. **A**: Hematoxylin and eosin staining shows amorphous deposits in the subepithelial region (asterisk). The overlying epithelium is degenerated, and Bowman’s layer is completely replaced by deposits. Underneath these amorphous deposits, there are globular deposits of various sizes with irregular peripheral margins that stained weakly with eosin (arrow). **B**: Amyloidal deposition is confirmed in the subepithelium with Congo red staining (asterisk). While the globular deposits of various sizes located primarily in the anterior stroma stained negatively with Congo red (arrow) (original magnification 100×).

The removed corneal buttons from both eyes exhibiting identical pathological findings. The corneal epithelium showed variable thickness stained with hematoxylin and eosin. The basement membrane and Bowman’s layer were absent in the central part of the corneal button. An extracellular, amorphous, eosinophilic material was observed just beneath the epithelium. The subepithelial deposits stained positively with Congo red, showing an apple green birefringence, typical of amyloid under polarized light. Underneath and among these amorphous deposits, globular deposits with various sizes were found (Figure 4A). The latter was ovoid with irregular peripheral margins that stained weakly with eosin, and stained red with Masson trichrome. These globules located primarily in the anterior stroma and stained negatively with Periodic acid Schiff and Congo red (Figure 4B). No inflammatory cells could be seen around these deposits.

### Molecular analysis

**Figure 5:**
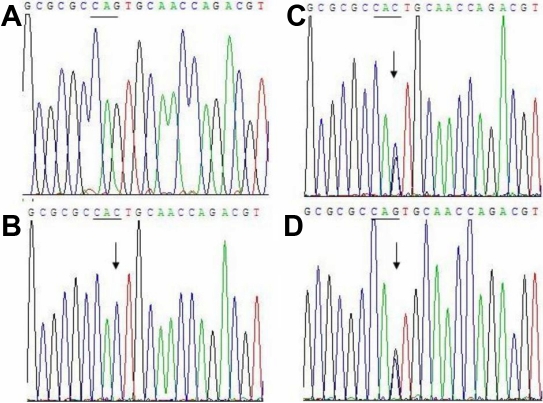
Sequence analysis of *TACSTD2* near codon 118. **A**: Normal sequence in a healthy control. **B**: The sequence from the proband shows the novel mutation c.354G>C (Q118H) in comparison to the normal control sequence. **C**: Double-wave peaks are seen at condon118 in his father. **D**: Double-wave peaks are seen at condon118 in his mother. Arrowheads indicate position of the mutation.

Analysis of the sequence data from the patient revealed a homozygous mutation c.354G>C in *TACSTD2* (Figure 5B). The nucleotide substitution of *TACSTD2* would result in replacement of Glutamine by Histidine (Q118H) at codon118.This mutation was found to be heterozygous in his father and mother and was confirmed by analysis of the reverse sequence data (Figure 5C,D). No mutations were detected in healthy controls (Figure 5A).

## Discussion

To the best of our knowledge, it is the first report showing gelatinous drop-like corneal dystrophy (GDLD) combined with spheroidal degeneration with clinicopathological findings and mutation analysis.

GDLD has four distinct subtypes in the literature: band keratopathy type, stromal opacity type, kumquat-like type and typical mulberry type [2]. Spheroidal degeneration has been classified into three clinical types [8]. Type 1 occurs bilaterally in the cornea without evidence of other ocular disease. Type 2 or secondary spheroidal degeneration is associated with underlying ocular pathology, and type 3 is conjunctival type with or without attendant corneal involvement. A familial category was added later by Meisler to explain the presence of central corneal spheroidal degeneration in multiple family members beginning in childhood [9]. Therefore, spheroidal degeneration is not just a degenerative process, but also may occur, rarely, as a corneal dystrophy [10].

The case we presented here manifested as spheroidal degeneration clinically at the first visit. The patient was young, with no history of other ocular disease, trauma or environmental exposure, which indicated primary spheroidal degeneration. He developed typical manifestations of GDLD (mulberry type) combined with spheroidal degeneration two years after the superficial keratectomy. The pattern of subepithelial location of the amyloid deposits and the mutation analysis of *TACSTD2* confirmed the diagnosis of GDLD, and the pathological properties of the spheroidal droplets were similar to those observed by other authors [11].

Secondary corneal amyloidois has been abserved in a variety of corneal and ocular diseases such as trichiasis, trachoma, lepra, sarcoidosis, interstitial keratitis, phlyctenular keratitis, uveitis, chronic post-traumatic inflammation, glaucoma, and keratoconus [12]. According to the mutation analysis, we considered that the amyloidosis in our patient was primary. However, the gelatinous change became obvious after superficial keratectomy, and the absence of a positive family history and consanguinity in our patient was uncommon.

An association between amyloidosis and spheroidal keratopathy has been recorded in the literature. Mashima et al. [13] reported a case of primary band-shaped spheroidal degeneration with subepithelial amyloid deposit secondary to keratoplasty. Santo et al. [14] described a case of primary spheroidal degeneration associated with subepithelial corneal amyloidosis clinically and histopathologically. Secondary amyloidosis and primary spheroidal corneal degeneration was suggested in these two studies, but no confirmation. Akiya et al. [15] reported a case of gelatinous drop-like corneal dystrophy in one eye and band-shaped spheroidal corneal degeneration in the other eye. The patient was a member of Japanese family with gelatinous drop-like corneal dystrophy. But no histopathological and mutation analysis was done. It was uncertain if gelatinous change was combined with spheroidal degeneration or not.

The point at issue in this case is what kind of deposits came first, the droplets or the amyloid, or did they appear simultaneously? It seemed that spheroidal droplets came first according to the slit-lamp examination, and pathological examination of the specimen taken after superficial keratectomy at the first visit revealed no amyloid deposits (data not showed). However, we could not exclude the possibility that amyloidosis do exist because of the limits of specimen. But at least, it was clear that gelatinous drop-like change became more obvious after the first surgery. We speculate that surgery may compromise the junction between the epithelial cells which is abnormal in GDLD patients and increase permeability of corneal epithelium, making the amyloid deposits aggregation worse [16].

To date, there are no published data on the recurrence of spheroidal degeneration among patients treated with keratoplasty, while GDLD has high recurrence rate after surgery [17]. After the penetrating keratoplasty, we could found that both spheroid droplets and the gelatinous drop-like deposits recurred, which may indicate the dystrophy characteristics of spheroid degeneration in this case.

*TACSTD2* consists of a single exon spanning about 1.8 kb of genomic DNA, and codes for a protein of 323 amino acids [3]. The 40-kDa TACSTD2 protein contains an epidermal growth factor-like repeat, a thyroglobulin repeat, a transmembrane domain, and a phosphatidylinositol-binding site harboring phosphorylatable serine and threonine residues near the COOH terminus [3]. The mutation Q118H detected in this study located in the thyroglobulin repeat, which is conserved sequence of *TACSTD2* and is considered to encode a functionally important part of the protein [18]. Q118H would lead to amino acid substitutions within *TACSTD2* and may change the function of it.

Up to now, approximately 25 mutations of *TACSTD2* have been found [18-21]. The most frequent mutation reported is 118 Q→X. It seems codon 118 is a hot spot of *TACSTD2* mutations. Considerable phenotypic variation has been reported [2]. It is the first report of GDLD combined with spheroidal degeneration with a novel missense mutation of *TACSTD2* (Q118H). More cases and study are needed to determine whether it is a new subtype or just a phenotypic variation of GDLD.
